# The consumption of culinary preparations and ultra-processed food is associated with handgrip strength in teenagers

**DOI:** 10.1186/s12937-022-00818-5

**Published:** 2022-10-22

**Authors:** Susana Cararo Confortin, Aline Rodrigues Barbosa, Bianca Rodrigues de Oliveira, Elma Izze da Silva Magalhães, Maylla Luanna Barbosa Martins Bragança, Maria Teresa Seabra Soares de Britto e Alves, Renata Bertazzi Levy, Rosângela Fernandes Lucena Batista, Poliana Cristina de Almeida Fonseca Viola, Antônio Augusto Moura da Silva

**Affiliations:** 1 Collective Health Post-Graduate Program, Federal University of Maranhão, Rua Barão de Itapari, 155, 65020-905, São Luís, Maranhão, Brazil; 2 School of Sports, Federal University of Santa Catarina, Florianópolis, Santa Catarina, Brazil; 3 Department of Preventive Medicine, School of Medicine, University of São Paulo, São Paulo, Brazil; 4 Department of Nutrition, Federal University of Piauí, Teresina, Piauí Brazil

**Keywords:** Muscular strength. Muscle Strength Dynamometer. Food processing. Food intake. Eating. Adolescent

## Abstract

**Background:**

A nutrient-poor and hypocaloric diet may be associated with lower handgrip strength (HGS), whereas a high-quality or balanced diet may be associated with higher HGS. However, no study has used the NOVA system for classifying food by their degree of processing.

**Objective:**

To analyze the association between food consumption according to the degree of food processing and HGS in Brazilian teenagers.

**Methods:**

This cross-sectional study included teenagers aged 18 and 19 years old from the 1997/98 São Luís’ birth cohort, Maranhão, Brazil. HGS (kilogram-force) was measured via a Jamar Plus + dynamometer. Food consumption was assessed using a semiquantitative food frequency questionnaire. The energy intake of culinary preparations (unprocessed or minimally processed food and processed culinary ingredients), processed, and ultra-processed foods was evaluated in percentages and categorized in tertiles. The associations between each food group intake and HGS was estimated via crude and adjusted linear regression models. A directed acyclic graph was used to identify confounding factors.

**Results:**

We evaluated 2,433 teenagers, 52.1% of which were girls. For boys, adjusted analysis showed an association between the highest HGS and the 3^rd^ tertile of culinary preparation consumption (β: 1.95; 95%CI: 0.80; 3.10) and between the lowest HGS and the 3^rd^ tertile of ultra-processed food consumption (β: -2.25; 95%CI: -3.40; -1.10). Among girls, the consumption of culinary preparations in the 3^rd^ tertile was associated with higher HGS (β: 0.76; 95%CI: 0.05; 1.46).

**Conclusions:**

Higher consumption of culinary preparations and lower consumption of ultra-processed foods can contribute to reduce the chance of lower HGS in adult life. Interventions to promote the development and preservation of muscle strength should include dietary recommendations.

## Introduction

Handgrip strength (HGS) measurement is a noninvasive, highly valid, and reliable assessment of health, mortality [1], and muscle strength in different age groups, including teenagers [2–4].

Age, gender, body measurements, laterality [5], physical activity [6], and food consumption [7–10], among others, may influence HGS. Consumption of micronutrient-poor and hypocaloric food may be associated with decreased HGS [9]; and a high-quality [11] or balanced diet [10], with a higher one. In fact, nutrition can affect muscle mass, strength, and physical performance [12].

Although recent studies have investigated this correlation in teenagers [7–10], none have investigated the association between HGS and food intake according to the NOVA food classification system groups [13].

NOVA classifies foods according to their nature, purpose, and degree of processing into the following groups: unprocessed or minimally processed, processed culinary ingredients, processed, and ultra-processed. All four groups in the NOVA classification include some form of processing. The processes and ingredients used in ultra-processed foods, however, make them nutritionally unbalanced, ready-to-eat, hyperpalatable, and highly profitable, hence promoting ultra-processed foods in place of other food groups [13].

Thus, since HGS is a health, morbidity, and mortality indicator, identifying its modifiable factors may guide preventive interventions to reduce adults’ health problems. Therefore, this study aims to analyze the association between food consumption, according to their degree of processing, with HGS in teenagers from the 1997/98 São Luís Birth Cohort.

## Methods

### Study design and population

This cross-sectional study was conducted in 2016 and is nested in the 1997/98 São Luís Birth Cohort, carried out in the capital of Maranhão State, in Northeastern Brazil [14].

Our baseline assessment (March 1997 to February 1998) consisted of systematically sampling 1/7 of births in 10 maternity hospitals (both public and private) in São Luís. Births outside of hospitals, in hospitals with less than 100 deliveries/year, and outside São Luís were excluded, providing a total sample of 2,493 live births.

In total, 673 children aged seven to nine years old were evaluated in 2005/2006, and again, in 2016 (*n* = 687). This follow-up included 1,828 teenagers born in São Luís in 1997 who had not participated in the original cohort. These teenagers’ mothers answered a fundamental part of the perinatal questionnaire. Inclusion of new individuals was twofold: by random sampling of the information for 1997 in the Brazilian Live Births Information System (SINASC) and by identifying volunteers in schools and universities. That stage of the study encompassed 2,515 teenagers. However, it only gathered data from 2,433 participants, since 82 of them had no HGS information [14].

### Data collection

The structured instrument for data collection took the form of face-to-face interviews and we used Research Electronic Data Capture (REDCap) [15] to register and manage data.

### Dependent variable

HGS (kilogram-force – Kgf) was measured via a Sammons Preston’s Jamar Plus + dynamometer, adjusted to individuals’ hand size. The test [16] required individuals to be seated with their feet on the ground, tested arm in a 90° flexion, forearm in a neutral position, palm facing up, exerting as much force as possible on the dynamometer [16]. Although both arms were measured three times, only interviewees’ dominant hand mean strength, with one-minute breaks in between attempts (Kgf), was considered.

### Independent variables

Food consumption was assessed using a food frequency questionnaire (FFQ), developed by Schneider et al. [17] and validated for teenagers in São Luís [18]. The FFQ collected information on food intake during the 12 months prior to interviews. Food intake was evaluated and grouped according to the NOVA degree of processing classification: culinary preparations (unprocessed or minimally processed foods, and processed culinary ingredients), processed, and ultra-processed culinary ingredients [percentage of caloric participation] [13].

The FFQ was comprised of 106 items whose average intake frequency was obtained via eight response options: never or less than once/month; one to three times/month; once/week; two to four times/week; five to six times/week; once/day; two to four times/day; and equal to or more than five times/day [19]. Photos of average food portions were made available for viewing on a computer to improve that measurement. Whether teenagers’ portions were of the same size (average), larger (1.5 the average) or smaller (0.5 the average) [17] than the photographed ones was also recorded. The *Tabela para Avaliação do Consumo Alimentar em Medidas Caseiras* (Food Intake in Homemade Measures Evaluation Table [20]) was use to convert average homemade portions into grams or milliliters.

We multiplied daily food intake (in grams or milliliters) by recorded portion sizes to estimate the energetic contribution of each NOVA food group [18]. The *Tabela Brasileira de Composição de Alimentos* (Brazilian Table of Food Composition – TACO) [21], the *Tabela de Composição Nutricional dos Alimentos Consumidos no Brasil* (Nutritional Composition Table of Foods Consumed in Brazil) [22], the USDA’s nutrient database for standard reference [23], and product label information were assessed to estimate the nutrient values of a 100 g or milliliters of each food/preparation. The daily energetic intake of each food item and their sum total (i.e., of all FFQ items) were estimated, allowing us to estimate each food group energetic contribution by their total calories.

The energetic contribution percentages of culinary-preparation (any and all food prepared with unprocessed or minimally processed foods), processed, and ultra-processed food groups were stratified into low (< 1st tertile), middle (≥ 1st tertile and < 3rd tertile) and high (≥ 3rd tertile). Following our tertiles: i) culinary preparations, 1st tertile < 52.6%; 2nd tertile ≥ 52.6% and < 63.8 and, 3rd tertile ≥ 63.8%; ii) processed food, 1st tertile < 2.8%, 2nd tertile ≥ 2.8% and < 5.3%, and 3rd tertile (≥ 5.3); and iii); and ultra-processed food, 1st tertile < 27.6%, 2nd tertile ≥ 27.6% and < 38.9%, and 3rd tertile ≥ 38.9.

### Adjustment variables

The following variables were used: age (in full years), gender (male and female), skin color (white, Black, and mixed race – Indigenous and Asians were excluded due to their small numbers), years of education completed (0 to 8, 9 to 11 or 12 or more years), and socioeconomic class, according to the 2016 *Classificação Econômica Brasil* (Brazilian Criteria of Economic Classification—CCEB) [A, B (B1 + B2), C (C1 + C2), D/E, in which A is the richest, most schooled class; and D/E, the poorest and least schooled] [24]. The following variables were also used: working (yes/no), tobacco smoking (yes/no), and alcohol consumption [(yes/no) via the Alcohol Use Disorder Identification Test (AUDIT)] instrument [25]. The Mini-international neuropsychiatric interview – Brazilian version 5.0.0 – DSM IV) [26] (M.I.N.I. Questionnaire) was used to assess major depressive episodes or depression (yes/no).

The short version [27] of the International Physical Activity Questionnaire (IPAQ) was used to assess total physical activity: insufficiently active, < 300 min/per week; and physically active, ≥ 300 min/per week.

### Data analysis

Descriptive analyses with estimates of absolute frequencies and percentages were performed. The Wilcoxon test was used to compare outcome (HGS) means between groups.

Associations between food intake and HGS were analyzed via linear regression models with estimates for crude and adjusted linear regression coefficients and their respective 95% confidence intervals (95%CI). There was a correlation between gender and food intake, and analyses were stratified by gender.

**Fig. 1:**
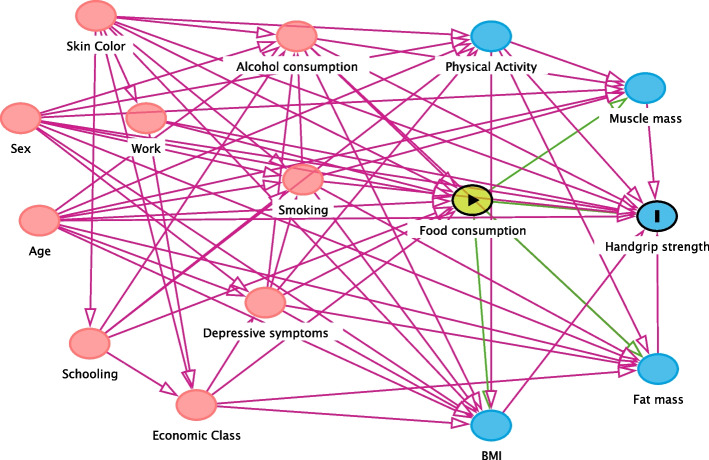
Directed acyclic graph of the associations between food intake and grip strength

A directed acyclic graph (DAG)—drawn in DAGitty 3.0 (Fig. 1)—was used to identify the minimum adjustment required for the confounder control [28]. The current literature was employed as the basis for elaborating the interrelations between food consumption and HGS. The variables selected for the backdoor criterion were age, socioeconomic status, skin color, work, alcohol consumption, tobacco smoking, total physical activity, and depressive symptoms. All analyses were performed using the statistical software Stata 13 (Stata Corp., College Station, USA).

### Ethical aspects

The second follow-up of the São Luís’ birth cohort 1997/98 was approved by the Research Ethics Committee of the University Hospital of the Federal University of Maranhão, under registry no. 1,302,489. Informed consent forms were signed by guardians. All projects meet the criteria in resolution no. 466/2012 of the Brazilian Health Council and its complementary regulations.

## Results

We analyzed 2,433 teenagers of all genders whose mean HGS values were 28 (± 9.4) Kgf. Boys’ mean values (35.2 ± 7.7) were higher than girls’ (21.4 ± 4.8) (< 0.001). According to Table 1, both boys and girls showed significant age; years of study completed; socioeconomic class; work; tobacco smoking; alcohol consumption; major recurrent depressive episodes; total physical activity; and culinary-preparation, processed, and ultra-processed food intake differences.

**Table 1:** Sample characterization according to gender, and handgrip strength (mean and standard deviation) according to adolescents’ demographic, socioeconomic, lifestyle, and food consumption characteristics. São Luís, Maranhão, Brazil, 2016/2017

	**Boys**		**Girls**			**HGS**		
						**Boys**	**Girls**	
	***n***	**%**	***">n***	**%**	***">p***	***">n***(%)	***">n***(%)	***">p***
**Age (years)**					0.008^†^			
18	843	49.6	855	50.4		34.9(7.8)	21.3(4.9)	≤ 0.001*
19	322	43.8	413	56.2		36(7.6)	21.7(4.8)	≤ 0.001*
**Years of education completed**					< 0.001^†^			
0 to 8	85	65.9	44	34.1		34.5(7.8)	20.7(4.3)	≤ 0.001*
9 to 11	1.010	47.3	1.124	52.7		35.4(7.7)	21.4(4.8)	≤ 0.001*
12 or more	64	40.8	93	59.2		34.0(6.9)	22.0(5.0)	≤ 0.001*
**Skin color**					0.397^†^			
White	219	45.3	264	54.7		34.7(7.3)	21.3(4.6)	≤ 0.001*
Black	190	47.6	209	52.4		35.3(8.0)	21.8(4.8)	≤ 0.001*
Mixed race	751	48.9	786	51.1		35.4(7.8)	21.4(4.9)	≤ 0.001*
**Socioeconomic Status**					< 0.001^†^			
A-B	324	50.8	314	49.2		35(7.2)	21.4(4.7)	≤ 0.001*
C	537	49.4	549	50.6		35.1(7.8)	21.4(4.9)	≤ 0.001*
D-E	161	37.0	274	63.0		36.2(8.1)	21.3(5.1)	≤ 0.001*
**Work**					< 0.001^†^			
No	947	46.2	1.101	53.7		34.9(7.6)	21.3(4.8)	≤ 0.001*
Yes	218	56.6	167	43.4		36.8(7.8)	22.0(5.0)	≤ 0.001*
**Tobacco smoking**					< 0.001^†^			
No	1.106	47.1	1.241	52.9		35.2(7.8)	21.4(4.9)	≤ 0.001*
Yes	59	68.6	27	31.4		35(5.9)	22(3.5)	≤ 0.001*
**Alcohol consumption**					< 0.001^†^			
No	622	44.0	791	56.0		35.6(7.9)	21.04(4.9)	≤ 0.001*
Yes	531	53.0	471	47.0		34.9(7.4)	21.5(4.7)	≤ 0.001*
**Recurrent major depressive episodes**					< 0.001^†^			
No	1.124	50.0	1.125	50.0		35.3(7.7)	21.4(4.8)	≤ 0.001*
Yes	41	22.3	143	77.7		32.2(8.3)	21.6(5.3)	≤ 0.001*
**Total Physical Activity**					< 0.001^†^			
Insufficiently active	478	35.9	852	64.1		34.1(7.7)	21.2(4.8)	≤ 0.001*
Physically active	671	62.3	406	37.7		36(7.5)	21.9(4.9)	≤ 0.001*
**Culinary preparations (%)**					0.026^†^			
1^st^ tertile	354	44.2	446	55.8		34.1(7.5)	21.2(4.4)	≤ 0.001*
2^nd^ tertile	390	48.6	413	51.4		35.2(7.3)	21.5(5.2)	≤ 0.001*
3^rd^ tertile	413	50.9	399	49.1		36.2(8.1)	21.6(4.9)	≤ 0.001*
**Processed (%)**					< 0.001^†^			
1^st^ tertile	340	41.9	471	58.1		34.7(7.9)	21.5(4.6)	≤ 0.001*
2^nd^ tertile	392	49.8	412	51.2		35.4(7.7)	21.6 (5.2)	≤ 0.001*
3^rd^ tertile	425	53.1	375	46.9		35.4(7.6)	21.1(4.7)	≤ 0.001*
**Ultra-processed (%)**					0.005^†^			
1^st^ tertile	418	51.9	391	48.1		36.2(8.1)	21.5(5.1)	≤ 0.001*
2^nd^ tertile	393	48.8	418	51.1		35.4(7.3)	21.5(5.0)	≤ 0.001*
3^rd^ tertile	346	43.0	449	57.0		33.8(7.5)	21.3(4.5)	≤ 0.001*

*Caption* Handgrip strength, *HGS*^†^ Chi-square test, *Wilcoxon test

Tables 2 and 3 show the crude and adjusted analyses of food intake, according to their degree of processing, compared to boys’ and girls’ grip strength, respectively. Crude data analysis showed greater HGS in boys with high culinary-preparation intake than those with lower intake, whereas those with high ultra-processed food intake showed lower HGS. Our adjusted analysis preserved this correlation: those with high culinary-preparation intake showed 1.95 Kgf more exerted strength (β: 1.95; 95%CI: 0.80; 3.10) than those with low intake. Boys with high ultra-processed food intake showed 2.38 Kgf less exerted strength (β: -2.38; 95%CI: -3.53; -1.23) than those with low intake (Table 2).

**Table 2:** Crude and adjusted analyses of food intake according to their degree of processing associated with handgrip strength in boys. São Luís, Maranhão, Brazil, 2016/2017

Characteristic	Crude analysis		Adjusted analysis*	
	**β (95%CI)**	***">p***	**β (95%CI)**	***">p***
**Food**				
**Culinary preparations (%)**		** < 0.001**		**0.001**
1^st^ tertile	1		1	
2^nd^ tertile	1.04(-0.06;2.15)		**1.37(0.20;2.54)**	
3^rd^ tertile	**2.09(1.00;3.18)**		**1.95(0.80;3.10)**	
**Processed (%)**		0.243		0.168
1^st^ tertile	1		1	
2^nd^ tertile	0.70(-0.42;1.82)		0.50(-0.67;1.68)	
3^rd^ tertile	0.68(-0.42;1.78)		0.80(-0.33;1.95)	
**Ultra-processed (%)**		** < 0.001**		** < 0.001**
1^st^ tertile	1		1	
2^nd^ tertile	-0.73(-1.79;032)		-0.32(-1.42;0.78)	
3^rd^ tertile	**-2.50(-3.59;-1.40)**		**-2.38(-3.53;-1.23)**	

*Caption* *Age, socioeconomic status, skin color, work, alcohol consumption, tobacco smoking, physical activity, and depressive symptoms

**Table 3:** Crude and adjusted analyses of food intake, according to their degree of processing, associated with handgrip strength in girls. São Luís, Maranhão, Brazil, 2016/2017

Characteristic	Crude analysis		Adjusted analysis*	
	**β (95%CI)**	***">p***	**β (95%CI)**	***">p***
**Culinary preparations (%)**		0.186		**0.035**
1^st^ tertile	1		1	
2^nd^ tertile	0.35(-0.30;1.00)		0.59(-0.10;1.29)	
3^rd^ tertile	0.44(-0.22;1.09)		**0.76(0.05;1.46)**	
**Processed (%)**		0.225		0.091
1^st^ tertile	1		1	
2^nd^ tertile	0.04(-0.60;0.69)		0.01(-0.67;0.69)	
3^rd^ tertile	-0.43(-1.09;0.23)		-0.63(-1.35;0.07)	
**Ultra-processed (%)**		0.571		0.341
1^st^ tertile	1		1	
2^nd^ tertile	-0.11(-0.56;0.79)		0.04(-0.67;0.76)	
3^rd^ tertile	-0.18(-0.83;0.48)		-0.34(-1.05;0.37)	

*Caption* *Age, socioeconomic status, skin color, work, alcohol consumption, tobacco smoking, physical activity, and depressive symptoms

Adjusted analyses showed that girls with high culinary-preparation intake had greater HGS (β: 0.76; 95%CI: 0.05; 1.46) than those with low intake (Table 3).

## Discussion

To the best our knowledge, this is the first study to investigate associations between HGS and food intake according to the NOVA classification system. Results showed higher HGS values in boys than in girls, which is consistent with the previous literature [10]. For boys, higher consumption of culinary preparations was associated with higher HGS, whereas those with high consumption of ultra-processed food were associated with lower HGS. On the other hand, we found an association of high culinary-preparation intake with higher HGS in girls. The associations found are plausible, considering that the nutritional composition of the food items in these food groups can favor anabolism and muscle development or degradation [8, 29], consequently altering HGS values.

Boys’ higher HGS, regardless of socioeconomic characteristics, reinforces the role of sexual dimorphism in body composition [10]; along with the effects of boys’ more active lifestyles [10].

Among the culinary-preparation subgroups consumed by higher-HGS boys, we noted the greater intake of highly biologically valued proteins (red meat, chicken/poultry, and eggs) and vitamin C (fruits). Girls showed the highest intake (in calories and grams) of culinary preparations, in addition to a higher intake of fruit, chicken, and poultry (data not shown). While meat and eggs are sources of protein that favor protein anabolism, fruits are rich in vitamins and have anti-oxidative and anti-inflammatory effects, which also promote muscle strength [30].

The association between higher culinary-preparation intake and higher HGS in boys and girls converges could be explained, in part, by data from previous studies [9, 11, 31]. Longitudinal data in Ng et al.^11^ showed an association between protein intake and HGS only in boys, suggesting that higher protein intake may lead to higher HGS. The quality and quantity of the protein ingested and teenagers’ apparent greater sensitivity to dietary protein anabolism than adults [29] could explain such an association.

In Kang et al. [9], higher-HGS teenagers had “balanced” dietary patterns— characterized by greater whole grain, vegetable, fruit, alga, and dairy product intake (26.6 kg ± 0.7) than those with “ready-to-eat” (25.3 kg ± 0.2) and Western-style fast-food dietary patterns (25.8 kg ± 0.4). Ng et al. [8], however, found no association between measured dietary components and girls’ HGS; true only for boys.

In this study, high ultra-processed food intake was associated with lower HGS in boys. These results converge, in part, with Kang et al.’s study, in which teenagers with a Western-style fast-food dietary pattern—containing several ultra-processed items—showed a lower HGS than those with “balanced” diets. However, after adjusting for confounding effects, these associations failed to maintain themselves [9].

A previous study with the same sample as this investigation found lesser muscle mass in teenagers with high ultra-processed diets, which is probably due to lower protein and higher carbohydrate (highly glycemic and fatty) intake [32]. The ultra-processed foods consumed in this study were fast foods, sugary drinks, sausages, and instant noodles among male teenagers with lower HGS (data not shown). For girls, the non-association between the consumption of ultra-processed foods and HGS can relate to the fact that the HGS tertile mean values, according to the consumption of ultra-processed foods, were extremely close. Therefore, these characteristics seem to not affect female HGS.

The exact mechanism behind a greater ultra-processed food intake and HGS remain unknown. Nonetheless, obesity might mediate this correlation since studies have shown that the greater consumption of these items increases the risk of overweight [33]. Adiposity can affect muscle ability, predisposing one to decreased muscle strength and potentially endangering the balance between fat and muscle mass or strength [4, 34].

### Limitations

Since this is a cross-sectional study, it is incapable to precisely establish the temporality between exposure and outcome, making it impossible to infer causality in this association. The use of self-reported measures for skin color, socioeconomic class, family income, and physical activity may lead to information biases, even though we obtained them via validated instruments applied by trained interviewers. The FFQ may overestimate food consumption, but it considers usual diet as a more important exposure factor than punctual consumption and is thus preferable to evaluate exposure intensity [35]. Moreover, NOVA neither rated nor contained all the food items found in our sample’s diets. In those cases, we used information from the *Pesquisa de Orçamentos Familiares* (Research on Family Budgets—POF) 2017/18 for the state of Maranhão.

### Strengths

This study evaluated HGS, adopted instruments and procedures from studies involving different populations (including teenagers), and constructed a conceptual theoretical model—DAG— to identify confounding factors and adjust analyses, avoiding spurious associations and estimation errors [36].

## Conclusion

This study shows the possible association of higher culinary-preparation intake with higher HGS and that of high ultra-processed food with lower HGS in male teenagers. It also found an association between high culinary-preparation intake and higher HGS in female teenagers. Thus, promoting healthy diets via the consumption of culinary preparations and reducing ultra-processed food intake may stimulate HGS maintenance or increase—an indicator of general health—and may have repercussions on greater functional capacity and fewer morbidities throughout life.

## Data Availability

The data supporting the findings of this study are available via the email rosangela.flb@ufma.br but restrictions apply to the availability of these data, which were used under license for the current study and so, are not publicly available. Data are, however, available from the authors upon reasonable request and with Rosangela Fernandes Lucena Batista’s permission.

## Abbreviations

HGS: Handgrip strength
SINASC: Brazil Live Births Information System
REDCap: Research electronic data capture
FFQ: Food frequency questionnaire
TACO: Brazilian table of food composition
AUDIT: Alcohol Use Disorder Identification Test
CCEB: Economic Classification Brazil
M.I.N.I.: Mini International Neuropsychiatric Interview
IPAQ: International Physical Activity Questionnaire
95%CI: 95% Confidence intervals
DAG: Directed Acyclic Graph
Research on Family Budgets – POF: Pesquisa de Orçamentos Familiares
